# Thrombospondin-1 signaling through CD47 inhibits cell cycle progression and induces senescence in endothelial cells

**DOI:** 10.1038/cddis.2016.155

**Published:** 2016-09-08

**Authors:** Qi Gao, Kexin Chen, Lu Gao, Yang Zheng, Yong-Guang Yang

**Affiliations:** 1 The First Hospital and Institute of Immunology, Jilin University, Changchun, China; 2 Columbia Center for Translational Immunology, Columbia University College of Physicians and Surgeons, New York, NY, USA

## Abstract

CD47 signaling in endothelial cells has been shown to suppress angiogenesis, but little is known about the link between CD47 and endothelial senescence. Herein, we demonstrate that the thrombospondin-1 (TSP1)-CD47 signaling pathway is a major mechanism for driving endothelial cell senescence. CD47 deficiency in endothelial cells significantly improved their angiogenic function and attenuated their replicative senescence. Lack of CD47 also suppresses activation of cell cycle inhibitors and upregulates the expression of cell cycle promoters, leading to increased cell cycle progression. Furthermore, TSP1 significantly accelerates replicative senescence and associated cell cycle arrest in a CD47-dependent manner. These findings demonstrate that TSP1-CD47 signaling is an important mechanism driving endothelial cell senescence. Thus, TSP1 and CD47 provide attractive molecular targets for treatment of aging-associated cardiovascular dysfunction and diseases involving endothelial dysregulation.

Endothelial cell (EC) senescence is accompanied with vascular dysfunction, including arterial stiffening and remodeling,^1^ impaired angiogenesis,^2, 3^ reduced endothelial repair capability and increased incidence of cardiovascular disease.^4, 5, 6^ Cellular senescence can occur *in vivo* or *in vitro* in response to various stressors,^7, 8, 9, 10^ leading to suppression of cell proliferation. EC senescence has been reported to contribute to the pathogenesis of age-associated vascular diseases, such as atherosclerosis.^11^ Thus, further understanding the mechanisms of EC senescence may help to identify effective targets for antisenescence therapy and treatment aging-associated cardiovascular disorders.

Previous studies have shown that the secreted matricellular protein thrombospondin-1 (TSP1) is as potent inhibitor of angiogenesis^12^ and its antiangiogenic activity is mediated by its receptors, CD36^13, 14^ and CD47.^15, 16^ CD47 is a ubiquitously expressed transmembrane protein that serves as a ligand for signal regulatory protein-*α* and is a signaling receptor of TSP1. The TSP1-CD47 pathway has an important role in several fundamental cellular functions, including proliferation, apoptosis, inflammation and atherosclerotic response.^17^ Ligation of CD47 by TSP1 has been shown to inhibit nitric oxide (NO)/cGMP signaling in vascular cells, leading to suppression of angiogenic responses.^16^ Recently, it was reported that lack of CD47 expression in ECs may enable these cells to spontaneously gain characteristics of embryonic stem cells.^18^ However, the potential role of CD47 in regulation of EC senescence has not been well explored. The present study was initiated to determine the role and mechanisms of TSP1-CD47 signaling pathway in regulating cell cycle progression and replicative senescence of ECs.

## Results

### CD47 deficiency promotes EC proliferation

**Figure 1:**
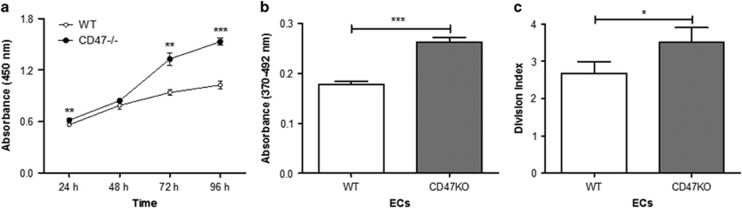
CD47 deficiency promotes EC proliferation. (**a**) WT *versus* CD47^−/−^ EC expansion measured by CCK8. (**b**) EC proliferation determined by measuring BrdU incorporation. (**c**) Division index of WT *versus* CD47^−/−^ ECs measured by CFSE dilution. Data shown are a representative of three independent experiments with similar results (mean±S.D.). *P<0.05, **P<0.01 and ***P<0.001

Primary ECs were prepared from the brain of 8-week-old wild-type (WT) or CD47^−/−^ mice, cultured *in vitro* for 4 days, and then passaged and used immediately (at passage 2 (P-2)) in the assays. First, we assessed the number of viable cells daily using the Cell Counting Kit-8 (CCK8). Although the number of viable cells were comparable between WT and CD47^−/−^ EC cultures until day 2, the latter thereafter yielded significantly more viable cells (Figure 1a). Moreover, CD47^−/−^ ECs showed significantly increased bromodeoxyuridine (BrdU) incorporation (Figure 1b) and carboxyfluorescein succinimidyl ester (CFSE) dilution (Figure 1c) compared with WT ECs. Taken together, these results demonstrated an increased potential of CD47^−/−^ ECs to proliferate and expand compared with WT ECs.

### CD47 deficiency promotes angiogenesis *in vitro* and *in vivo*

**Figure 2:**
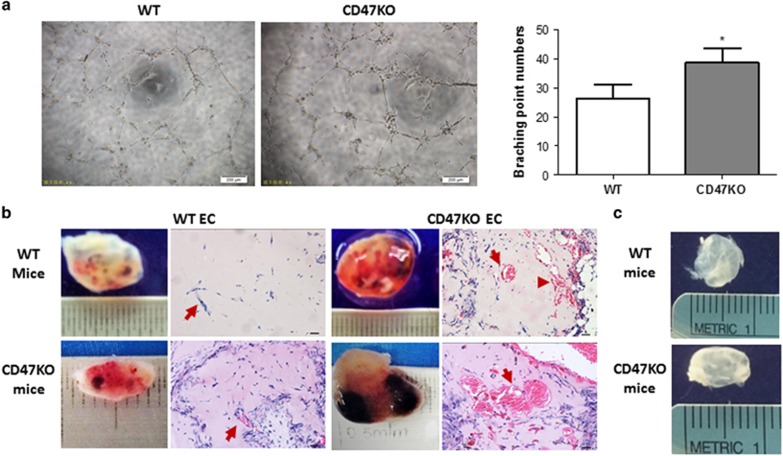
CD47 deficiency promotes angiogenesis. (**a**) Comparison of *in vitro* angiogenic potential of WT *versus* CD47^−/−^ ECs (at P-2) by tube formation assay. Shown are representative images (left; scale bar, 200 *μ*m) and numbers of the branching points per field (right; shown are mean±S.D. of three independent experiments; *P<0.05). (**b** and **c**) The potential of *in vivo* angiogenic potential of WT *versus* CD47^−/−^ ECs (at P-2) estimated by Matrigel plug assay. Shown are gross morphology and histology (HE; scale bars, 20 *μ*m) of representative Matrigel plugs. (**b**) Matrigel plugs with WT (left panels) or CD47^−/−^ (right panels) ECs from WT (top panels) and CD47^−/−^ (bottom panels) mice. (**c**) Control plugs without ECs from WT (top) and CD47^−/−^ (bottom) mice. Arrows point to vessels. Each group consisted of three animals and the experiments were repeated two times

*In vitro* angiogenic potential of WT *versus* CD47^−/−^ ECs (at P-2) was assessed by EC tube formation assay. CD47^−/−^ ECs showed a significant increase in endothelial tube length and branch point numbers compared with WT ECs (Figure 2a). We also compared the *in vivo* angiogenic potential of WT and CD47^−/−^ ECs using the Matrigel plug assay, in which EC-containing Matrigels were subcutaneously implanted into WT or CD47^−/−^ mice, and the plugs removed 2 weeks later for analysis. Matrigel plugs with CD47^−/−^ ECs, harvested from both WT and CD47^−/−^ mice, showed significantly increased microvessel density compared with Matrigel plugs with WT ECs (Figure 2b), demonstrating that CD47 deficiency can promote angiogenesis *in vivo*. The observed neovascularization was attributed to the injected ECs, as microvessel formation was not detected in Matrigel plugs without ECs in either WT or CD47^−/−^ mice (Figure 2c). Interestingly, both CD47^−/−^ and WT ECs showed increased neovascularization potential, as shown by higher microvessel density, when injected into CD47^−/−^ mice compared with WT mice, indicating that the environment of CD47^−/−^ mice may enhance angiogenesis. Taken together, these results indicate that CD47 deficiency in both ECs and the mouse microenvironment may promote angiogenesis.

### CD47 deficiency delays EC senescence and promotes cell cycle progression

**Figure 3:**
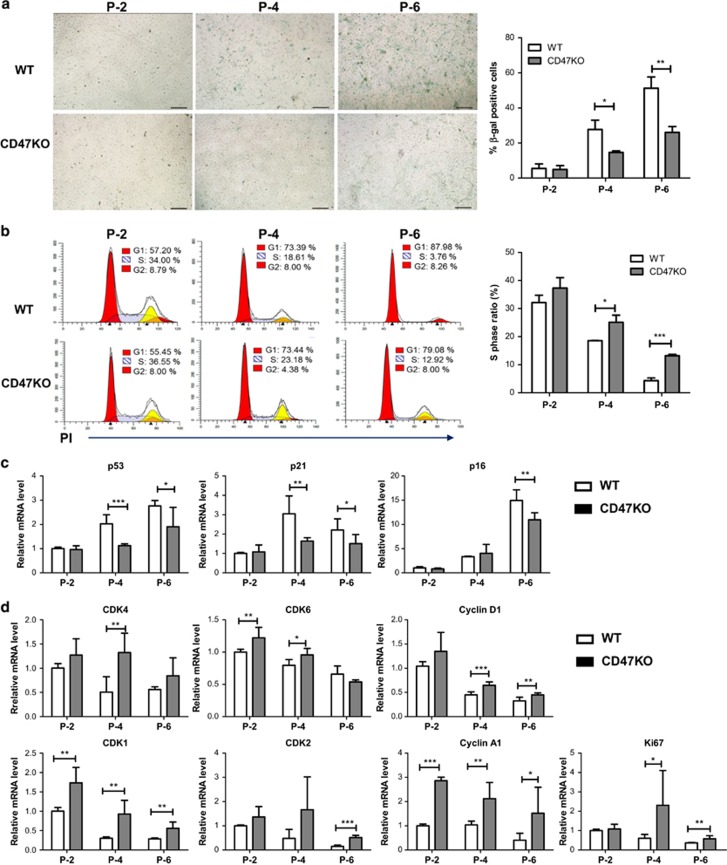
CD47 induces EC senescence and inhibits cell cycle progression. (**a**) Representative images of SA-*β*-gal staining (left; senescent cells are stained green) and percentages (right; mean±S.D.; *n*=6) of senescent cells in WT *versus* CD47^−/−^ ECs at the indicated passages. Data from a representative of three independent samples are shown. *P<0.05 and **P<0.005. (**b**). Cell-cycle analysis was performed with PI staining by flow cytometry. Shown are representative flow cytometric profiles (left panel) and percentages (right; mean±S.D.; *n*=6) of cells at the S phase (calculated using the Modfit Software, Topsham, ME, USA) in WT *versus* CD47^−/−^ ECs at the indicated passages. *P<0.05 and ***P<0.001. (**c and** **d**). Relative mRNA expression levels (mean±S.D.; *n*=6) of cell cycle regulators in WT *versus* CD47^−/−^ ECs at the indicated passages. *P<0.05, **P<0.005 and ***P<0.001

To determine the role of CD47 in EC senescence, we measured senescence-associated *β*-galactosidase (SA-*β*-gal) activity, a representative feature of senescence, in ECs during continuous cultivation. Both WT and CD47^−/−^ ECs showed a clear increase in SA-*β*-gal^+^ cells overtime; however, the increase was markedly less in the latter group and the percentages of SA-*β*-gal^+^ cells in CD47^−/−^ ECs at P-4 and P-6 were significantly lower when compared with WT ECs at the same passages (Figure 3a).

One of the most important features of senescence is cell cycle arrest, which is an indispensable marker for identifying the senescence of cells *in vivo* and *in vitro*.^19^ Cell cycle distribution was analyzed by measuring cell DNA content. The proportion of cells in the G1 phase increased with passage, as reflected by the decreased ratio of S phases in both WT and CD47^−/−^ ECs. However, the percentages of cells in the S phase were significant greater in CD47^−/−^ ECs compared with those in WT ECs (Figure 3b).

We further analyzed mRNA expression levels of the key cell cycle regulators. We first compared the mRNA expression levels of key cell cycle inhibitors (CKIs) p53, p21 and p16, which are known to be upregulated in senescent cells.^20, 21^ Consistent with the cell cycle analysis results (Figure 3b), the expression of CKIs p53, p21 and p16 was significantly reduced in CD47^−/−^ ECs compared with WT ECs during the course of replicative senescence (Figure 3c). Accordingly, CD47^−/−^ ECs expressed a significantly higher levels of cell cycle promoters, including cyclin-dependent kinase 4 (CDK4), CDK6 and Cyclin D1. Cyclin D1 and CDK4/6 coregulate cell cycle progression by phosphorylating and thereby inhibiting retinoblastoma (RB),^22, 23^ which allows E2F-mediated S-phase gene transcription, such as CDK1, CDK2, Cyclin A, Cyclin E1 and Ki67.^24, 25^ Taken together, these results demonstrated a critical role for CD47 in the regulation of EC cell cycle progression and senescence.

### TSP1-CD47 signaling inhibits angiogenesis and induces EC senescence

**Figure 4:**
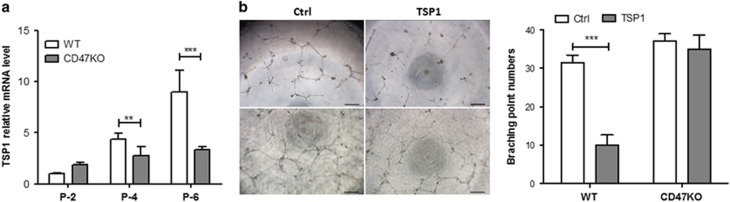
TSP1 inhibits EC angiogenic potential in CD47-dependent manner. (**a**) Levels (mean±S.D.; *n*=6) of TSP1 mRNA in WT and CD47^−/−^ ECs at the indicated passages. (**b**) Effect of TSP1 on angiogenesis determined by tube formation assay. Shown are representative images (left; scale bar, 200 *μ*m) and the number of branching points per field (mean±S.D.; *n*=3) in WT (top panel) and CD47^−/−^ (bottom panel) P-2 ECs. Data shown are from one representative experiment of two. **P<0.005 and ***P<0.001

Because CD47 is an important receptor for TSP1-induced inhibition of angiogenesis,^11^ we measured the levels of TSP1 production in EC cultures. WT ECs showed a significantly faster rate of increase and higher concentration in the level of TSP1 mRNA expression overtime compared with CD47^−/−^ ECs (Figure 4a). To further determine the role of TSP1-CD47 signaling in EC function, we compared the effect of exogenous TSP1 on the angiogenic potential of WT and CD47^−/−^ ECs at P-2, in which neither of these ECs produced significant amount of TSP1 (Figure 4a). Addition of TSP1 significantly inhibited the angiogenic potential of WT, but not CD47^−/−^, P-2 ECs (Figure 4b).

**Figure 5:**
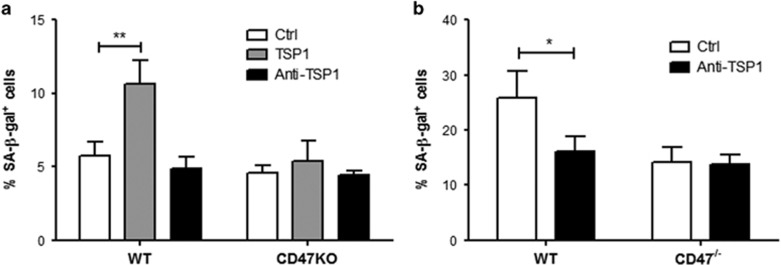
TSP1 induces EC senescence in CD47-dependent manner. WT and CD47^−/−^ ECs were cultured alone (Ctrl), or with TSP1 or anti-TSP1 monoclonal antibody (mAb) for 48 h, and the levels of senescent cells were determined by SA-*β*-gal staining. Shown are percentages (mean±S.D.; *n*=3) of WT and CD47^−/−^ ECs at P-2 (**a**) or P-4 (**b**). *P<0.05 and **P<0.005

Addition of TSP1 also led to a significant increase in the ratio of SA-*β*-gal+ senescent cells in the cultures of WT, but not CD47^−/−^, ECs (at P-2; Figure 5a). Addition of neutralizing anti-TSP1 antibodies had no effect on WT P-2 EC senescence (Figure 5a), likely due to the low level of TSP1 (Figure 4a), but mediated significant protection against senescence in WT P-4 ECs (Figure 5b). However, anti-TSP1 treatment had no detectable effect on either P-2 or P-4 CD47^−/−^ ECs (Figures 5a and b). Taken together, these results indicate that TSP1 signaling through CD47 not only inhibits angiogenesis but also induces EC senescence.

**Figure 6:**
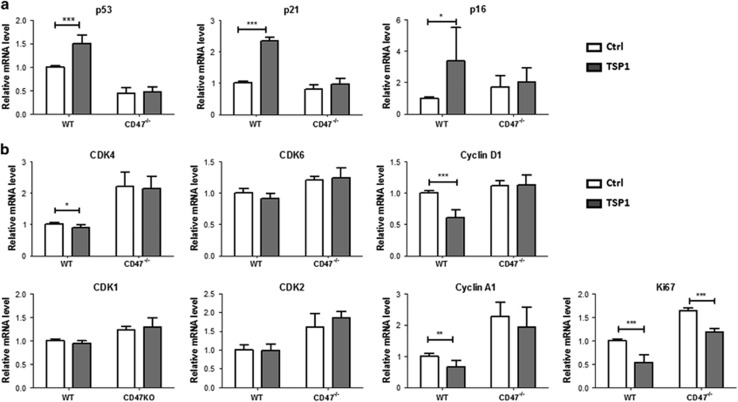
TSP1 inhibits cell cycle progression in CD47-dependent manner. (**a** and **b**) WT and CD47^−/−^ ECs at P-2 were treated with 1 *μ*g/ml TSP1 for 48 h and quantitative PCR (qPCR) was performed as described in the Materials and Methods section. Shown are mRNA levels (mean±S.D.; *n*=6) of CKIs (**a**) and promoters (**b**). *P<0.05, **P<0.005 and ***P<0.001

TSP1 also inhibits cell cycle progression in ECs. Addition of TSP1 to WT EC cultures led to a significant upregulation of CKIs p53, p21 and p16 (Figure 6a) and downregulation of cell cycle promoters CDK4, Cyclin D1 and Cyclin A1 (Figure 6b). Interestingly, in CD47^−/−^ EC cultures, TSP1 did not significantly affect the expression of most cell cycle regulators measured, although there was a reduction in Ki67 expression (Figure 6). Although the observed effects of TSP1 on ECs were predominantly mediated by CD47, this result suggests that some of the effects might be CD47-independent.

## Discussion

Senescence is an accumulation of various types of damage that lead to permanent cell growth arrest.^26^ Both reduced cellular replicative capacity and impaired angiogenesis are markers of EC senescence. Senescent cells are irreversibly arrested in the G0/G1 phase of the cell cycle and lose the ability to response to growth factors. Senescence in brain microvascular ECs is closely related with stroke and vascular dementia.^27^ It has been reported that loss of CD47 protects neurons from both caspase-dependent and -independent apoptosis.^28^ In the present study, we found that TSP1-CD47 signaling inhibits EC proliferation and function, and induces EC replicative senescence. ECs lacking this signaling pathway, as shown in CD47-deficient cells, showed markedly improved cell cycle progression, delayed replicative senescence and improved angiogenic potential. Thus, the TSP1-CD47 signaling pathway has a critical role in EC senescence and function.

In addition to the intrinsic properties of ECs, the microenvironment of CD47-deficient mice is far superior to that of WT mice in supporting angiogenesis. We observed that both CD47^−/−^ and WT ECs showed increased neovascularization potential when injected into CD47^−/−^ mice compared with WT mice. However, further studies are needed to address the mechanism(s) by which CD47 deficiency in the mouse microenvironment promotes angiogenesis.

TSP1 is known to be a potent inhibitor of angiogenesis.^12^ Previous studies have shown that the antiangiogenic activity of TSP1 is mediated by its receptors CD36^13, 14^ and CD47.^15, 16^ A recent study reported that lack of TSP1-CD47 signaling in ECs enables the cells to acquire the potential for self-renewal and stemness by increasing c-myc expression.^18^ We found that during *in vitro* culture, WT ECs showed no significant reduction in CD47 expression (Supplementary Figure 1) but more rapid increase in TSP1 production compared with CD47^−/−^ ECs, which is consistent with the previously reported increase of TSP1 levels in senescent tissues.^29^ However, the delayed senescence for CD47^−/−^ ECs is unlikely to be due to lower TSP1 levels, as addition of exogenous TSP1 failed to accelerate CD47^−/−^ EC senescence. These results demonstrate that CD47, but not CD36, is required for TSP1-induced EC senescence. These findings are in agreement with previous observations that CD47 is critical for the antiangiogenic activity of TSP1 at the physiological concentration.^16^ However, CD36 might also be involved in TSP1-mediated inhibition of EC function, as a significant reduction in Ki67 expression was detected in CD47^−/−^ ECs when cultured with exogenous TSP1.

Cyclin D1 regulates the G1/S transition by binding and activating CDK4 and CDK6, whereas CKIs including a p53 target gene p21 and p16 inhibit the kinase activity of the cyclin/CDK complex. P21 and p16 induce RB dephosphorylation and inhibit the RB/E2F pathway, leading to G1-phase arrest. Our data showed that during the process of replicative senescence, TSP1-CD47 signaling induces activation of CKIs and inhibition of cyclins and CDKs, leading to cell cycle arrest in ECs. Despite a significant delay, senescence and the associated inhibition of cell cycle progression was also seen in CD47^−/−^ ECs during *in vitro* culture. Taken together, these results indicate that EC senescence can be induced by both TSP1-CD47-dependent and -independent mechanisms, although the contribution of the former is clearly highly significant. Thus, TSP1 and CD47 are attractive therapeutic targets for antiaging therapy and the treatment of EC dysfunction-associated diseases, such as stroke and cancer.

## Materials and Methods

### Animals

WT and CD47^−/−^ mice on the C57BL/6J (B6) background were obtained from the Jackson Laboratory (Bar Harbor, ME, USA). Protocols involving animal experiments were approved by the Subcommittee on Research Animal Care of the First Hospital of Jilin University, and all of the experiments were performed in accordance with the protocols.

### EC isolation and culture

Primary mouse brain microvascular ECs were isolated and purified according to a previously published protocol,^30^ and used immediately. The freshly isolated ECs were cultured in M131 medium supplemented with 5% MVGS (Gibco-Invitrogen, MA, USA). The cells were passaged at confluency every 4 (for P-1–3) or 6 (for P-4–6) days. The purity was determined by flow cytometer with anti-mouse CD31 antibody (BD Bioscience, Franklin Lakes, NJ, USA) and was >98%.

### Cell proliferation assay

ECs at P-2 were used for assessing cell proliferation by the CCK8 assay, BrdU incorporation assay or CFSE dilution assay. For the CCK8 assay, a CCK8 Cell Proliferation Kit (Beyotime, Beijing, China) was used according to the manufacturer's instructions. ECs were seeded into a 96-well plate at 3 × 10^3^ cells per well with 100 *μ*l medium and cultured in a incubator at 37 °C with 5% CO_2_, and CCK8 solution was added (10 *μ*l per well) 2 h before measuring the absorbance at 450 nm. BrdU assay was performed using Roche BrdU ELISA Kit (Roche Diagnostics, Basel-Stadt, Switzerland) as per the manufacturer's instructions. ECs were seeded at 1 × 10^4^ per well and cultured for 12 h in a humidified incubator at 37 °C with 5% CO_2_. The absorbance was measured at 370 nm, with 492 nm as the reference wavelength. Absorbance was measured using a microplate reader (BioTek, Winooski, VT, USA). For CFSE assay, ECs were stained with CFSE at a final concentration of 2 *μ*M (in PBS with 0.1% BSA) for 10 min at 37 °C, and washed with ice-cold DMEM with 0.1% FBS. Cell proliferation was determined by measuring CFSE dilution using flow cytometry (BD FACS Canto II) and presented as division index.

### Endothelial tube formation assay

Matrigel (Corning, Kennebunk, ME, USA) was added into a 96-well plate (50 *μ*l per well) and allowed to polymerize for 30 min at 37 °C. ECs were seeded at 1 × 10^4^ per well and grown in M131 supplemented with 5% MVGS for 24 h in a incubator at 37 °C with 5% CO_2_. The images were acquired 6 h after incubation. Branching points in three randomly selected fields (x4 magnified) were counted and data are presented as the average number of branching points per field.

### Matrigel plug assay

Matrigel plug assay was used for estimating angiogenesis *in vivo*. Briefly, 5 × 10^6^ WT or CD47^−/−^ ECs were mixed with Matrigel (BD Bioscience), and injected subcutaneously into WT or CD47^−/−^ mice. After 2 weeks, the Matrigel plugs were removed and analyzed by immunohistochemistry. Briefly, Matrigel plugs were fixed in 10% neutral-buffered formaldehyde and embedded in paraffin, and then sectioned at a thickness of 3 *μ*m. Hematoxylin and eosin staining was performed to observe the vessels.

### Quantitative real-time PCR

Total RNA was extracted with Trizol (Invitrogen, Waltham, MA, USA), and cDNA was synthesized using TransScript First-Strand cDNA Synthesis SuperMix (TransGen Biotech, Beijing, China). Quantitative real-time PCR was performed using a SYBR Green Kit (TransGen Biotech) with a StepOnePlus Real-Time PCR System (Applied Biosystems, Inc., Carlsbad, CA, USA), and the primer sets used for p21, p53, p16, CDK1, CDK2, CDK4, CDK6, Cyclin D1, Ki67 and *β*-actin (Sangon Biotech, Shanghai, China) are listed in Table 1. Relative gene expression was normalized to *β*-actin.

### SA-*β*-gal activity analysis

SA-*β*-gal activity was determined using a Senescence *β*-Galactosidase Staining Kit (Beyotime) according to the manufacturer's instructions. ECs at different passages were plated in a 12-well plate (6 × 10^4^/well) and analyzed when reaching 80–90% confluency. In some experiments, 1 *μ*g/ml recombinant TSP1 (R&D Systems, Inc., Minneapolis, MN, USA) or 1 *μ*g/ml TSP1 antibody (Abcam, Cambridge, MA, USA) was added. SA-*β*-gal-positive cells (ie, senescent cells) were identified as green-stained cells under standard light microscopy, and the frequencies of senescent cells were determined by counting ~800 cells in three random fields.

### Cell cycle analysis

ECs at different passages were harvested by trypsinization when reaching 80–90% confluency. Cells were washed two times with PBS, fixed at −20 °C in 70% ethanol for 12 h and stained in 300 *μ*l propidium iodide (final concentration of 50 mg/ml) and 0.1% Triton X-100 at 37 °C for 15 min. The distribution of cells in different phases of the cell cycle was analyzed by flow cytometry (BD FACS Canto II).

### Statistical analysis

All data are presented as mean±S.D., and the significance was calculated by the Student's *t*-test. Differences with *P*<0.05 were considered statistically significant.

## Figures and Tables

**Table 1:** qPCR primers for p21, p53, p16, CDK1, CDK2, CDK4, CDK6, Cyclin D1, Ki67 and *β*-actin

	**Forward (5′–3′)**	**Reverse (5′–3′)**
Mouse *β*-actin	CGCCACCAGTTCGCCATGGA	TACAGCCCGGGGAGCATCGT
Mouse Cyclin D1	CAGAAGTGCGAAGAGGAGGTC	TCATCTTAGAGGCCACGAACAT
Mouse p53	TCACAGCGTCTGTTGACATTT	AACAAGCTCATTACCCTGACA
Mouse p21	GTGGGTCTGACTCCAGCCC	CCTTCTCGTGAGACGCTTAC
Mouse p16	CGTACCCCGATTCAGGTGAT	TTGAGCAGAAGAGCTGCTACGT
Mouse CDK1	AAGAGCAAAATCCGTCCCTAGC	TCATCTCAACGAAGATACAGCCA
Mouse CDK2	GCGACCTCCTCCCAATATCG	GTCTGATCTCTTTCCCCAACTCT
Mouse CDK4	CTGAACCGCTTTGGCAAGAC	GCCCTCTCTTATCGCCAGAT
Mouse CDK6	GGCGATCCCACAGAAACCATA	AGGTAAGGGCCATCTGAAAACT
Mouse TSP1	TTGCCAGCGTTGCCA	TCTGCAGCACCCCCTGAA
Mouse Cyclin A1	GAGGTTCCTGACTTATCAGTGC	CCTTGATGACGTTGCTTTCCT
Mouse Ki67	CGCAGGAAGACTCGCAGTTT	CTGAATCTGCTAATGTCGCCAA

Abbreviations: qPCR, quantitative PCR; TSP-1, thrombospondin-1

## Abbreviations

BrdU: bromodeoxyuridine
CCK8: Cell Counting Kit-8
CDK: cyclin-dependent kinase
CFSE: carboxyfluorescein succinimidyl ester
CKI: cell cycle inhibitor
HE: hematoxylin and eosin
RB: retinoblastoma
SA-*β*-gal: senescence-associated *β*-galactosidase
TSP1: thrombospondin-1
WT: wild type
