# Supplementary material for: Thrombospondin-1 signaling through CD47 inhibits cell cycle progression and induces senescence in endothelial cells
Source: Cell Death Dis. 2016 Sep 8;7(9):e2368–. doi: 10.1038/cddis.2016.155 (PMC5059850; doi:10.1038/cddis.2016.155)
Supplement: Supplementary Figure 1 [file cddis2016155x1.pdf]

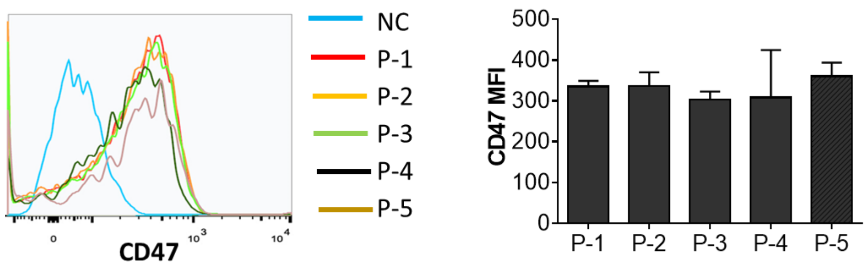

Figure S1. CD47 expression on WT ECs at different passages. WT ECs were cultured and harvested at the indicated passages for flow cytometry analysis of CD47 expression. Shown are representative flow cytometry profiles and the mean fluorescence intensities (MFI; ±SD). One of two experiments with similar results is shown. NC, native control.
